# Anxiety disorders alter cognitive-motor integration during visuomotor adaptation and retention

**DOI:** 10.1007/s00221-026-07304-y

**Published:** 2026-05-11

**Authors:** Leo Barzi, Matt Wilson, Christopher M. Hill

**Affiliations:** 1 School of Kinesiology, Louisiana State University, 2210 Huey P. Long Fieldhouse, Baton Rouge, LA 70803 USA; 2 School of Allied Health and Communicative Disorders, Northern Illinois University, Dekalb, IL USA

**Keywords:** Anxiety disorders, Motor preparation, Movement-readiness potentials, Visuomotor adaptation, Cognitive-motor integration, EEG

## Abstract

Anxiety disorders are associated with prefrontal dysfunction, yet their impact on neural mechanisms underlying skilled motor learning remains poorly understood. We examined movement-readiness potentials (MRPs) using electroencephalography during a visuomotor adaptation task in 31 young adults (13 with clinically diagnosed anxiety disorders, 18 controls). MRPs were analyzed across three temporal components: early motor preparation (− 1500 to − 500 ms), late motor preparation (− 500 to − 100 ms), and movement execution (− 100 to + 100 ms). Individuals with anxiety disorders showed significantly reduced MRP amplitudes during late motor preparation (*p* = 0.033) and movement execution (*p* = 0.047) compared to controls, while early motor preparation remained intact. Despite these neural alterations, both groups demonstrated equivalent behavioral performance, with similar learning and retention of a visuomotor rotation task. Anxiety disorders selectively disrupt late-stage cognitive-motor integration processes during movement preparation and execution. The dissociation between impaired neural activity and preserved behavioral performance suggests compensatory mechanisms that maintain motor learning despite underlying neural inefficiencies. These findings reveal that anxiety affects integrated systems of cognition and action, providing new insights into their functional neurophysiological impact.

## Introduction

Anxiety is a negative emotion driven by perceived uncertain or distant threats, triggering defensive behaviors like heightened vigilance(Grogans et al. 2023; Akiki et al. 2024). Excessive, prolonged anxiety results in anxiety disorders where individuals overestimate risks and persistently perceive threats in non-threatening situations (Thibaut 2017). Anxiety disorders are the most common psychological disorders, with 34% lifetime prevalence in the United States (Szuhany and Simon 2022). Beyond their high prevalence, anxiety disorders impose substantial human and economic burdens, including reduced quality of life, occupational impairment, and increased healthcare utilization (Hoffman et al. 2008). Anxiety disorders are a precursor and common comorbidity with neurological disorders, hindering treatment and rehabilitation. For instance, anxiety disorders can precede Parkinson’s disease by twenty years and triple dementia risk (Shiba et al. 2000; Savica et al. 2010; Burke et al. 2018).

Although anxiety disorders have traditionally been linked to dysfunction within prefrontal and amygdala-prefrontal circuitry (Bishop 2007; Shin and Liberzon 2010), recent evidence demonstrate cerebellar and primary motor cortex dysfunction also contribute to anxiety manifestation. The cerebellum, traditionally viewed as a motor coordinator, plays important roles in emotional processing and cognitive functions (Schmahmann 2019), and emerging evidence implicates cerebellar circuits in fear and anxiety-related disorders (Moreno-Rius 2018). Therapeutic effects of transcranial magnetic stimulation targeting motor areas further support motor system involvement in anxiety (Cirillo et al. 2019). These findings implicate a wider network of anxiety-related dysfunction that encompasses the motor system (Martins et al. 2024). Anxiety has been linked to impaired reaction time (Feldman et al. 2019), task accuracy (Lo et al. 2019), and movement coordination (Bastian 2008). However, the current body of literature has primarily evaluated acute anxiety, and less emphasis has been placed on anxiety disorders. Chronic anxiety may result in sustained alterations to prefrontal-motor connectivity that acute stressors cannot capture.

These findings point toward anxiety creating deficits in cognitive-motor integration, a bidirectional interaction of cognitive functions and motor control systems that produce purposeful movement behaviors (Rogojin et al. 2019). Cognitive-motor integration can be indexed using movement-readiness potentials (MRPs) recorded with electroencephalography (EEG) (Shibasaki and Hallett 2006; Alexander et al. 2016; Schurger et al. 2021). Specifically, MRPs are recorded in the contralateral motor cortices as negative deflection in neural activity prior to voluntary movement (Shibasaki and Hallett 2006). MRPs have been associated with activation of supplementary motor area, presupplementary motor area, and primary motor cortex, but not exclusively. MRPs also reflect unconscious determinants of voluntary decisions that emerge several seconds before conscious awareness (Libet et al. 1982, 1983; Soon et al. 2008). Decrements in the MRPs have been found in patients with cerebellar degeneration (Tarkka et al. 1993; Wessel et al. 1994) and schizophrenia (Westphal 2003; Donati et al. 2021), suggesting a wide range of cortical and subcortical inputs. Moreover, neuroeconomics studies have found changes to MRP amplitude are directly related to human decision making (Maoz et al. 2019; Triggiani et al. 2023; Verbaarschot et al. 2025). Given MRPs’ sensitivity to both decisions and sensorimotor processes, this opens the possibility it can index changes associated with anxiety disorders.

Visuomotor adaptation is a widely recognized skill learning assessment that utilizes motor and cognitive areas (i.e., motor cortex, cerebellum, and prefrontal cortex) (Taylor and Ivry 2011, 2014; Krakauer et al. 2019; Reuter et al. 2022; Tsay et al. 2024). Participants adjust their reaching movement to a visual perturbation to hit a target. The gradual reduction of error over time by updating internal models (implicit) for upcoming movement and altering movement strategies (explicit) (Krakauer and Mazzoni 2011). The cerebellum and prefrontal cortex make distinct contributions to adaptation, with the cerebellum supporting implicit adaptation and the prefrontal cortex contributing to explicit strategy development and reinforcement learning (Taylor and Ivry 2014).The retention of the visual perturbation is often tested in conditions where visual feedback is withdrawn (i.e., no cursor or feedback) and participants are asked to move in the same way they responded to the perturbation. Encoding and recall of motor memories is attributed to activation of the motor cortex (Galea et al. 2011). Given the cortical and subcortical connections involved in visuomotor adaptation, this task is a strong model for understanding cognitive-motor integration and how it changes with various psychological disorders. For instance, patients with schizophrenia are unable to develop robust explicit strategies to counter visual perturbations and have reduced task generalization (Bansal et al. 2019). Previous studies have found interactions between prefrontal and motor cortical activation during the learning and recall of a visuomotor adaptation task (Reuter et al. 2022). For instance, increased activation of the anterior cingulate cortex decreased motor cortex output, as represented by decrements in MRP amplitude (Anguera et al. 2009; Hill et al. 2020, 2021).

Despite evidence that anxiety affects motor-related brain regions and that MRPs index motor preparation, no studies have directly examined how anxiety disorders alter the temporal dynamics of movement preparation during adaptive skill learning. Clarifying these mechanisms has important clinical implications, including treatment monitoring, identification of individuals at risk for comorbid motor dysfunction, and refinement of intervention targets. The purpose of this study was to examine how anxiety disorders alter neural mechanisms of motor preparation during visuomotor adaptation and retention. We hypothesized that individuals with anxiety disorders would exhibit (1) reduced MRP amplitudes during motor preparation and (2) behavioral deficits in visuomotor adaptation learning or retention compared to controls.

## Methods

All procedures were approved by the University Institutional Review Board and were conducted according to the principles expressed in the Declaration of Helsinki. Participants were recruited from the local population of the University and the surrounding communities using word of mouth, electronic announcements, and posted flyers. All participants provided written informed consent prior to participating. Thirty-one young healthy adults participated in this study [age range: 19–27 years, mean age ± standard deviation (SD): 21.59 ± 2.02 years, Edinburgh Handedness Inventory (EHI) (Oldfield, 1971) mean handedness score ± SD: 94.54 ± 6.93 [All right handed], males: 9, females: 22)]. Participants were free of major physiological (musculoskeletal, neurological, cardiovascular) disorders. 13 participants were clinically diagnosed with an anxiety disorder [generalized anxiety disorder: 11, social anxiety disorder: 1, generalized anxiety disorder and social anxiety disorder: 1]. Among those with an anxiety disorder, 11 were actively taking medication to manage their condition. All participants completed the State-Trait Anxiety Inventory (Spielberger and Sydeman 1994). An a priori power calculation was performed using a smallest effect size of interest (Lakens 2022) of Cohen’s f = 0.25, where α = 0.05 and β = 0.2 (i.e., statistical power = 80%). Based on these criteria, an adequate sample size of 24 was calculated. Our collected sample size (*n* = 31) is similar to a recent study (Mussini and Di Russo 2023) that explored differences in movement preparation brain activity and trait anxiety levels (*n* = 32). This study found differences in MRPs between high and low anxious participants with large effects.

### Experimental procedures

#### Visuomotor adaptation task acquisition

**Fig. 1:**
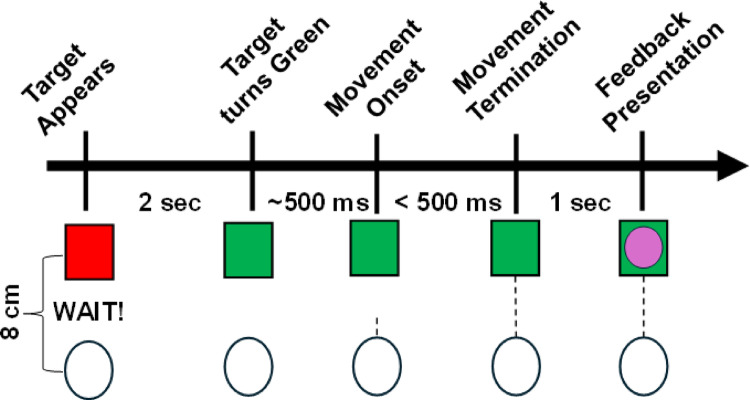
Single trial time course. Movement initiation was cued by the target. No cursor feedback will be provided during the reach (dashed line). A “WAIT” message will appear instructing the participant to pause until the target turns green, cueing them begin their reach. Endpoint feedback (pink dot) was provided at trial completion

The visuomotor task procedures followed those outlined in previous studies (Galea et al. 2015; Song and Smiley-Oyen 2017). The task was coded and performed in Matlab R2024b software (Mathworks, Inc.) using the Psychophysics Toolbox extensions (Brainard 1997; Kleiner et al. 2007; Pelli 1997). Participants were seated in front of an 83.6 cm computer monitor that was elevated to 26.5 cm using a custom-built stand and parallel to the table. A Wacom tablet was placed underneath the monitor. A Wacom pen embedded into a cone was used to perform the task. Participants were instructed to grasp the cone, similar to an air hockey paddle, and maintain the same grip throughout the experiment. Furthermore, participants were positioned close enough to the monitor so that their right hand was visually occluded. Trials were participant-initiated, by moving a cursor represented by a white dot into a small circle located in the center of the screen. Afterwards, a red target represented by a square was displayed 8 cm from the starting circle in eight different positions, pseudorandomly so that every set of eight consecutive trials would include one of each of the target positions. After a 2 s pause the target turned green and the participant was instructed to reach to the target as swiftly and accurately as possible (Fig. 1).

**Table 1:** Description of cursor rotation, feedback type, and number trials in each task condition that was performed by the participants

Task condition	Cursor rotation	Feedback type	Number of trials
Baseline	0°	Endpoint	100
Adaptation	45° CCW	Endpoint	200
No vision	45° CCW	No feedback	200
Washout	0°	Endpoint	100

CCW, counterclockwise

Cursor trajectory was not provided during the reach. End point feedback, represented by a pink dot, was provided at the termination of their reach. The removal of online visual feedback during the task was necessary to prevent feedback-related neural activity from confounding the MRP analysis. However, without online cursor visibility, participants could only learn via movement endpoint information. This might have encouraged the use of a more explicit strategy rather than implicit model-based learning. Participants were instructed to hit target with their reach so that the pink dot would cover green target. A duration criterion of 300 ms was placed on each trial, meaning that once participants initiated the trial, they had 300 ms to move their cursor past the invisible circle boundary that passes through the target circle, which is similar to previous studies (Galea et al. 2015; Song and Smiley-Oyen 2017). If the trial was not completed within 300 ms, an auditory message would play stating the words “too slow” and the participant would redo the trial. Participants performed a total of 600 trials consisting of four testing conditions: Baseline (100 trials), Adaptation (200 trials), Retention (200 trials), and Washout (100 trials) (Table 1). After every block of 50 trials, a rest period was provided, and participants were instructed to keep their arm under the visual occlusion. During the Baseline and Washout conditions, target and cursor movement were congruent. Adaptation featured an incongruent position of the cursor and the target, with the cursor trajectory, rotated 45° counterclockwise to the target, requiring the participant to adapt their movement to hit the target. Prior to the start of Adaptation, the participants were instructed to “Adapt your movement to hit the target”. Retention still featured a 45° counterclockwise rotation, but no end point feedback. Prior to the start of the retention stage, participants were instructed to “Maintain your adaptation despite not having cursor feedback.” For Baseline and Washout, participants were instructed to “Reach towards the target.” The NASA Task Load Index assessed the subjective cognitive workload after each task condition (Baseline, Adaptation, Retention). Participants answered six questions using a 21-point graduated scale representing different workload dimensions [Mental Demand, Physical Demand, Temporal Demand, Performance Success, Effort, and Frustration] (Hart and Staveland 1988; Hart 2006).

## EEG acquisition

Surface EEG data was recorded at 1000 Hz with a 32 channel actiCAP electrode system and a Brain Products LiveAmp EEG amplifier (Gilching, Germany). Electrodes were placed according to the 10–20 system at sites FZ, CZ, PZ, OZ, FP1, FP2, F3, F4, F7, F8, FT7, FT8, FC3, FC4, C3, C4, CP3, CP4, P3, P4, T3, T4, T7, T8, P7, P8, TP7, TP8, TP9, TP10, O1, and O2. A saline solution was applied with a blunt tip syringe into the individual electrodes to lower electrical signal noise. Electrical impedance for each electrode was kept below 10kΩ throughout the data collection.

## Visuomotor adaptation task analysis

Response time was defined in seconds, as the time between the target turning green and the hand’s movement 10 mm from the start position. The 10 mm threshold ensured that small involuntary or accidental movements noise did not prematurely trigger response time recording (Avraham et al. 2021).

Peak velocity was defined as the maximum absolute speed observed during each trial. Peak velocity was calculated by obtaining x and y velocity components from hand position data, which were combined to yield absolute hand speed (√(vx² + vy²)). Prior to this calculation, duplicate position samples (frames in which hand position did not change) were removed, and samples exceeding 10 times the interquartile range of successive differences were excluded as outliers. Peak velocity data were cleaned separately for each participant column. Trials were excluded if peak velocity exceeded an absolute cutoff of 2000 mm/s or if the value deviated more than 3 standard deviations from that participant’s mean.

Using a custom Matlab script, raw hand trajectories were resampled to include only movement-related samples and converted from pixels to millimeters. From these data, we computed trial-wise hand angle relative to the target direction [Hand theta (Θ)]. Sample to sample differences in both x and y coordinates were computed, and movement onset was identified as the earliest time point at which either coordinate deviated from the previous sample. Angular deviations were normalized using circular subtraction to account for the rotated visual feedback.

Learning stages were further defined over the time course of the task. Early Adaptation and Retention was defined as the average hand angle during the first 8 trials of each condition (Early Adaptation, Early Retention). Late Adaptation and Retention was defined as the average hand angle during the last 8 trials of each condition (Late Adaptation, Late Retention).

## NASA task load index analysis

Raw NASA Task Load Index scores for each dimension [Mental Demand, Physical Demand, Temporal Demand, Performance Success, Effort, Frustration]. An overall score was calculated for each participant following the procedures outlined by (Hart and Staveland 1988). These data were further divided into two subscales which combines scores across dimensions: Task-related [Mental, Physical, Temporal]; Behavior-related [Performance, Effort].

### EEG analysis

Raw EEG files were analyzed using the EEGLAB version 2024.0 (Delorme and Makeig 2004) in Matlab R2024b. The continuous data was preprocessed in the following steps: down sampled from 1000 Hz to 250 Hz, high pass filtered at 0.1 Hz, bad channels were interpolated, and channels were re-referenced to the common average. To examine movement preparation related neural activity, the data was segmented into time-locked data 2000ms epochs around the onset of movement, with a 1500 ms pre-movement baseline and a 500ms post-movement period. During epoching, baseline correction from 2000–1500ms was applied using amplitudes averaged across the premovement period. An initial visual inspection of the epochs was performed to remove trials containing excessive movement noise, electromyography, and blink artifacts. Signal decomposition was performed using independent components analysis on each participant’s data utilizing the ‘runica’ procedure in EEGLAB. Additional trials containing artifacts were identified using the IClabel tool in EEGLAB (Pion-Tonachini et al. 2019) and resultant artifact components of the signal decomposition and were removed from the analysis. Components reflecting eye blinks and electromyography activity were removed by visual inspection. Epoched EEG data for Adaptation and Retention conditions were each split into two halves with approximately 100 trials each to measure brain processes associated with the Early and Late stages of each condition, following methods used in previous studies (Anguera et al. 2009; Bracco et al. 2018; Labruna et al. 2019; Hill et al. 2021).

A region of interest approach was used to examine motor preparatory activity following methods from Hamel et al. (2018). Using custom EEGLAB scripts, the average movement readiness potential during each Condition (Adaptation, Retention) and Stage (Early, Late) was found for each subject across the F3, Fz, FC1, C3, and Cz electrodes. We selected this electrode cluster based on MRI studies that localized them to the midfrontal and motor regions of interest (Okamoto et al. 2004; Jurcak et al. 2007; Hamel et al. 2018). Following methods from (Jochumsen and Niazi 2020), mean amplitudes were calculated across three-time ranges that each represent different motor preparatory processes: the motor potential, the negative slope, and the readiness potential. The motor potential was calculated by taking the mean amplitude from − 100 to + 100 ms with 0 being the onset of movement and represents movement execution generated primarily through activity in the motor cortex (Shibasaki and Hallett 2006; Schurger et al. 2021). The negative slope was calculated by taking the mean amplitude from − 500 to − 100 ms and represents the translation of movement goals into detailed motor commands and is generated primarily through activity from the premotor and motor cortices (Jahanshahi et al. 1995; Praamstra et al. 1996; Cunnington et al. 2003). The readiness potential was calculated by taking the mean amplitude from − 1500 to − 500 ms and represents the earliest preparation for movement generated by the supplementary motor cortex (SMA), pre-SMA, and cingulate motor area that emerges before conscious awareness of movement (Kornhuber and Deecke 1965; Praamstra et al. 1996; Cunnington et al. 2003; Shibasaki and Hallett 2006). For reference, the target appeared approximately at − 2000 ms with 0 being the onset of movement.

### Statistical analysis

Participant descriptive data [age, handedness score, state anxiety inventory score, trait anxiety inventory score] were evaluated using separate independent samples student t-tests to determine differences between the anxiety disorder and control groups.

Hand angle derived from the visuomotor adaptation task was analyzed using a linear mixed-effects models to determine differences between Group (Anxiety, Control), Condition (Adaptation, Retention), and Stage (Early, Late). Hand angle was held as a dependent variable. Group, Condition, and Stage were held as fixed effects and individual subjects’ were held as random effects.

Subjective cognitive workload, as measured by the NASA Task Load Index dimensions, was assessed using separate linear mixed-effects models to determine differences between groups (Anxiety, Control) and conditions (Baseline, Adaptation, Retention). Overall NASA Task Load Index score and subscales scores [Task-Related, Behavior-Related] were held as dependent variables. Group and Condition were held as fixed effects and individual subjects’ were held as random effects.

Response time (seconds) after the target turned green was analyzed using a linear mixed-effects model to determine differences between Group (Anxiety, Control), Condition (Adaptation, Retention), and Stage (Early, Late). Response time was held as a dependent variable. Group, Condition, and Stage were held as fixed effects and individual subjects’ were held as random effects.

Peak velocity (mm/s) was analyzed using a linear mixed-effects model to determine differences between Group (Anxiety, Control), Condition (Adaptation, Retention), and Stage (Early, Late). Peak velocity was held as a dependent variable. Group, Condition, and Stage were held as fixed effects and individual subjects’ were held as random effects.

To examine the effects of Group (Anxiety, Control), Condition (Adaptation, Retention), and Stage (Early, Late) on movement readiness potentials amplitudes during the visuomotor adaptation task, three separate linear mixed-effects models were conducted on each component of the movement readiness potential (Boisgontier and Cheval 2016; Giboin et al. 2020; Kumari et al. 2020). ERP amplitudes were held as the dependent variables. Group, Condition, and Stage were held as fixed effects and individual subjects were held as random effects.

For all linear mixed model analysis, Satterthwaite approximation was used for degrees of freedom calculations to appropriately handle the mixed design structure. Individual slopes were not included due our small sample size and avoid convergence issues with an overly complex model (Burnham and Anderson 2004; Matuschek et al. 2017; Meteyard and Davies 2020). The advantages associated with linear mixed models, as opposed to conventional statistical methodologies, encompass the capability to account for measurements nested within individual subjects, the accommodation of missing and unbalanced data, prevention of information loss attributable to data averaging, and the facilitation of enhanced parameter estimation through the implementation of a partial pooling strategy (Boisgontier and Cheval 2016; Giboin et al. 2020; Kumari et al. 2020). Post-hoc pairwise comparisons were planned using estimated marginal means with Sidak adjustment to control for Type I error across multiple comparisons. All analyses were conducted using IBM SPSS Statistics (version 29.0).

## Results

The raw behavior and EEG data are available at https://osf.io/a65wk.

### Participant descriptive

To ensure similarity between groups and to confirm successful randomization we analyzed the demographic variables (Age, Handedness). Both groups were similar in age (t(29) = 1.180, *p*=.248, Cohen’s d (d) > = 0.429, mean differences (MD) = 0.863) and handedness scores (t(29) = 0.563, *p *= 0.578, *d *= 0.205, MD = 1.455). STAI is commonly used to index anxiety levels and has been associated with event related potentials in previous studies (Xia et al. 2017; Du et al. 2022; Mussini and Di Russo 2023; Zheng et al. 2024). As expected, we found that those with anxiety disorders had higher state (t(29) = 4.791, *p *< 0.001, *d* = 1.744, MD = 14.190) and trait anxiety inventory (t(29) = 3.926, *p *< 0.001, *d* = 1.429, MD = 16.010) scores compared to controls (Table 2).

**Table 2:** Participant descriptive data

Variable	Anxiety group (M ± SD)	Control group (M ± SD)	t(df)	*p*	Cohen’s d
n	13	18	–	–	–
Age (years)	22.3 ± 2.5	21.4 ± 1.6	1.1(29)	0.24	0.42
Handedness score (EDI)	93.1 ± 7.5	94.6 ± 6.8	0.5(29)	0.57	0.21
State Anxiety (STAI-S)	34.7 ± 7.9	20.5 ± 8.3	4.7(29)	< 0.001*	**1.7**
Trait Anxiety (STAI-T)	44.2 ± 10.9	28.2 ± 11.4	3.9(29)	< 0.001*	**1.4**

Presented as mean ± standard deviation.

*Represents a significant difference between anxiety and control

### Participant clinical characteristics

See Table 3.

**Table 3:** Participant clinical characteristics

Age	Sex	Diagnosis	Medication (Generic Name, Dose)	Drug class
26	Female	GAD + Social AD	Wellbutrin (Bupropion) 300 mg	DNRI
21	Female	GAD	None	–
20	Female	GAD	Zoloft (Sertraline) 50 mg	SSRI
21	Female	GAD	Effexor (Venlafaxine) 300 mg, Wellbutrin (Bupropion) 150 mg	SNRI, DNRI
27	Male	GAD	Propranolol 20 mg, Zoloft (Sertraline) 100 mg	Beta-blocker, SSRI
19	Male	Social AD	Bupropion 100 mg	DNRI
21	Female	GAD	Lexapro (Escitalopram) 20 mg	SSRI
24	Female	GAD	Bupropion 150 mg	DNRI
20	Female	GAD	Lexapro (Escitalopram) 20 mg	SSRI
24	Male	GAD	None	–
21	Female	Social AD	Paroxetine 25 mg	SSRI
24	Female	GAD	Prozac (Fluoxetine) 40 mg, Vraylar (Cariprazine) 1.5 mg	SSRI, Atypical antipsychotic
22	Female	GAD	Wellbutrin (Bupropion) 150 mg, Celexa (Citalopram) 20 mg	DNRI, SSRI

Psychiatric medications reported by participants in the anxiety disorders group

GAD, Generalized anxiety disorder; Social AD , Social anxiety disorder; SSRI, Selective serotonin reuptake inhibitor; SNRI, Serotonin-norepinephrine reuptake inhibitor; DNRI, Dopamine-norepinephrine reuptake inhibitor

#### Response time

Response time decreased from Early to Late stages and from Adaptation to Retention conditions across all participants. We found a significant main effect for Stage (F(1,87.000) = 27.726, *p* < 0.001, *d* = 0.62). Response time was faster during Late compared to Early [MD: 0.073, *p* < 0.001, 95% CIs = 0.045–0.101]. A significant main effect for Condition was also observed (F(1,87.000) = 26.696, *p* < 0.001, *d* = 0.52). Response time was faster during Retention compared to Adaptation [MD: 0.072, *p* < 0.001, 95% CIs = 0.044–0.099]. No significant main effect was found for Group (F(1,29) = 0.068, *p* = 0.796, *d* = 0.10). Similarly, no significant interactions emerged: Group × Stage (F(1,87.000) = 0.358, *p* = 0.551, *d* = 0.14), Group × Condition (F(1,87.000) = 1.294, *p* = 0.258, *d* = 0.24), Stage × Condition (F(1,87.000) = 0.066, *p* = 0.798, *d* = 0.06), or Group × Stage × Condition (F(1,87.000) = 0.000, *p* = 0.994, *d* = 0.09). These findings suggest that participants became faster in their responses as they progressed through practice (Late stage) and after learning was consolidated (Retention), with these changes occurring similarly across both anxiety and control groups.

### Peak velocity

Analysis of peak velocity revealed no significant differences between groups. Peak velocity decreased from Adaptation to Retention conditions across all participants, although no significant change was observed between Early and Late stages. We found a significant main effect for Condition (F(1, 87.000) = 16.207, *p* < 0.001, d = 0.19). Peak velocity was slower during Retention compared to Adaptation [MD = 50.39, *p* < 0.001, 95% CIs = 35.92–149.80]. No significant main effect was found for Stage (F(1, 87.000) = 0.467, *p* = 0.496, d = 0.02). Peak velocity was similar between Early and Late stages [MD = 6.45, *p* = 0.496, 95% CIs = − 50.47–63.36]. Similarly, no significant main effect was found for Group (F(1, 29) = 0.389, *p* = 0.538, d = 0.23). No significant interactions emerged: Group × Stage (F(1, 87.000) = 1.390, *p* = 0.242, d = 0.13), Group × Condition (F(1,87.000) = 2.119, *p* = 0.149, d = 0.16), Stage × Condition (F(1, 87.000) = 0.001, *p* = 0.973, d = 0.00), or Group × Stage × Condition (F(1,87.000) = 2.175, *p* = 0.144, d = 0.16). These findings suggest that participants’ peak velocity decreased as they moved from the adaptation to the retention phase, but this change, occurred similarly across both anxiety and control groups.

### NASA task workload

**Fig. 2:**
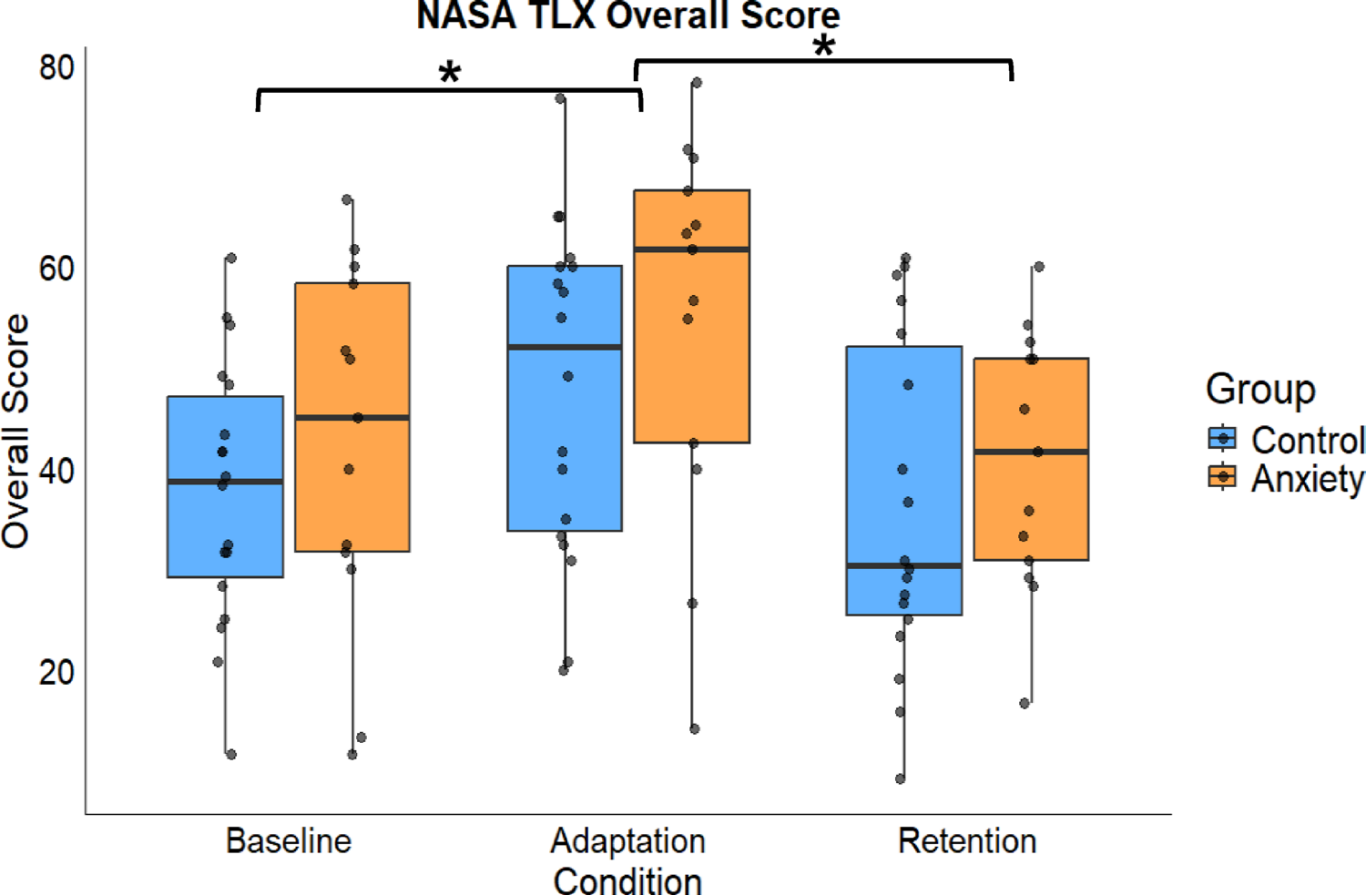
Total raw score of the NASA-TLX across conditions between anxiety and control groups. Error bars show standard error. * represents that adaptation had a significant difference from Baseline (*p* < 0.001) and Retention (*p* <  0.001)

Subjective cognitive workload changed across the motor adaptation conditions but not between groups. A significant main effect for Condition for overall cognitive workload (F(1,58.005) = 14.185, *p* < 0.001, *d* = 0.69). Overall cognitive workload increased in Adaptation compared to Baseline [MD: 11.230, *p* < 0.001, 95% CIs = 4.770–17.689, *d* = 0.69] and Retention [MD: 12.845, *p* < 0.001, 95% CIs = 6.385–19.304, *d* = 0.84]. Overall cognitive workload did not differ between Baseline and Retention [MD: 1.615, *p* = 0.903, 95% CIs = − 4.844–8.074, *d* = 0.16] (Fig. 2). No significant main effect was found for Group (F(1,29.001) = 1.214, *p* = 0.280, *d* = 0.50). No significant Group × Condition interaction emerged (F(1,58.005) = 0.117, *p* = 0.890, *d* = 0.26).

Task-Related cognitive workload demonstrated a similar pattern, where the introduction of the visuomotor rotation increased workload compared to other conditions. A significant main effect for Condition for overall cognitive workload (F(1,58.005) = 14.550, *p* < 0.001, *d* = 0.67). Task-Related cognitive workload increased in Adaptation compared to Baseline [MD: 11.829, *p* < 0.001, 95% CIs = 6.089–17.569, *d* = 0.68] and Retention [MD: 9.657, *p* < 0.001, 95% CIs = 3.917–15.397, *d* = 0.67]. Task-Related cognitive workload did not differ between Baseline and Retention [MD: 2.172, *p* = 0.733, 95% CIs = − 7.912–3.568, *d* = 0.01]. No significant main effect was found for Group (F(1,29.001) = 1.433, *p* = 0.241, *d* = 0.50). No significant Group × Condition interaction emerged (F(1,58.005) = 0.283, *p* = 0.755, *d* = 0.26).

This trend continued with Behavior-Related cognitive workload. A significant main effect for Condition for overall cognitive workload (F(1,58.005) = 5.034, *p* = 0.010, *d* = 0.52). Behavior-Related cognitive workload increased in Adaptation compared to Baseline [MD: 9.548, *p* = 0.029, 95% CIs = 0.737–18.330, *d* = 0.52] and Retention [MD: 10.061, *p* = 0.020, 95% CIs = 1.280–18.843, *d* = 0.53]. Behavior-Related cognitive workload did not differ between Baseline and Retention [MD: 0.513, *p* = 0.999, 95% CIs = − 8.269–9.294, *d* = 0.01]. No significant main effect was found for Group (F(1,29.001) = 0.351, *p* = 0.558, *d* = 0.50). No significant Group × Condition interaction emerged (F(1,58.005) = 1.111, *p* = 0.336, *d* = 0.35).

### Visuomotor task performance

**Fig. 3:**
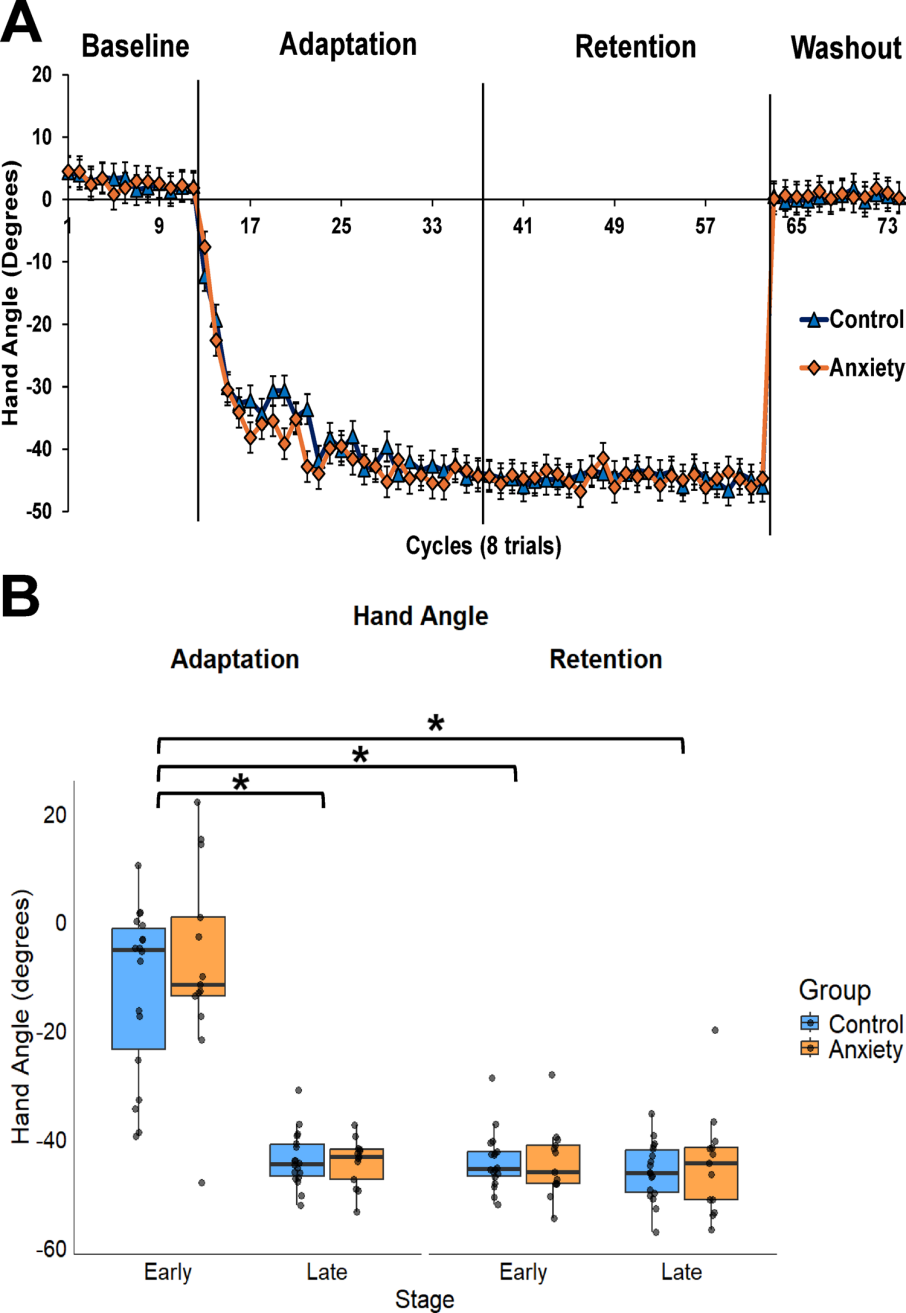
**A** Relative hand angle across epochs of eight trials all conditions between anxiety and control groups. 1 cycle represents the presentation of each target position (8 total). Represented as mean ± standard error. **B** Boxplot for the average hand angle across the Adaptation and Retention conditions for the Anxiety and Control groups during the Early and Late stages. Box ends represent the interquartile range (IQR) [25th percentile (Q1) -75th percentile (Q3)]. Whiskers represent minimum (Q1-1.5*IQR) and maximum (Q3–1.5*IQR). Median is represented by horizontal line inside of the box and dots represent individual data. **^** represents a significant difference compared to Early Adaptation

All groups adapted to the 45-degree rotation during Adaptation and retained it during Retention (Fig. 3A). We found a significant Stage x Condition interaction (F(1,87.005) = 102.576, *p* < 0.001, *d* = 1.10). Hand angle increased from Early Adaptation to Late Adaptation [MD: 34.139, *p* < 0.001, 95% CIs = 29.555–38.722, *d* = 2.46]. However, hand angle was similar during Early and Late Retention [MD: 1.107, *p* = 0.632, 95% CIs = − 3.476–5.691, *d* = 0.13]. Early Retention had a greater hand angle compared to Early Adaptation [MD: 34.3411, *p* < 0.001, 95% CIs = 29.727–38.894, *d* = 2.46]. Hand angle was similar between Late Adaptation and Late Retention [MD: 1.279, *p* = 0.581, 95% CIs = − 3.305–5.863, *d* = 0.19] (Fig. 3B). No significant main effects were found for Group (F(1,29.022) = 0.334, *p* = 0.568, *d* = 0.21). Similarly, no other significant interactions emerged: Group × Condition (F(1,87.005) = 0.232, *p* = 0.631, *d* = 0.26), Group × Stage (F(1,87.005) = 0.300, *p* = 0.585, *d* = 0.20), or Group × Condition × Stage (F(1,87.005) = 1.079, *p* = 0.302, *d* = 0.02).

### Movement readiness potentials

**Fig. 4:**
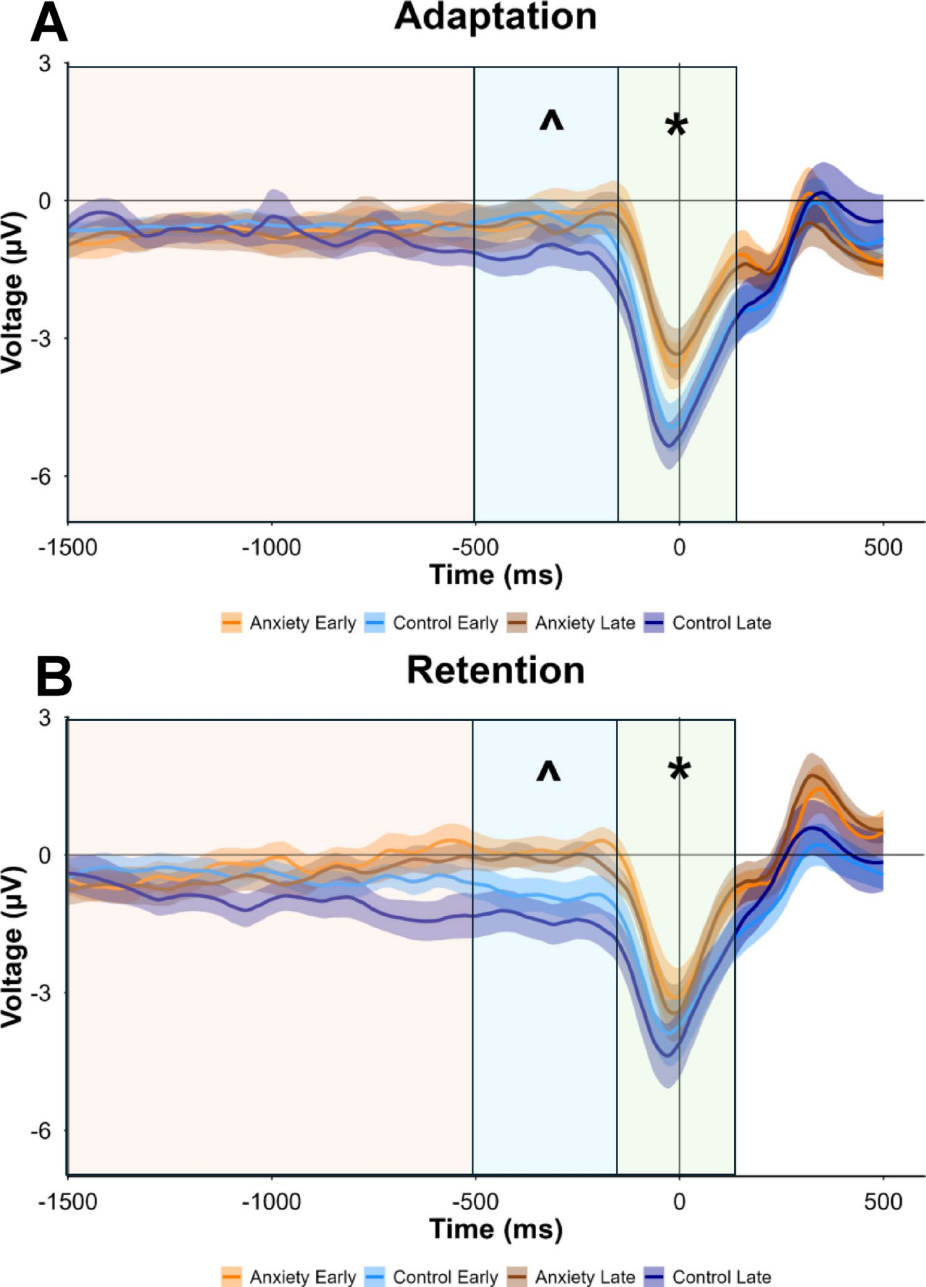
**A** Mean movement readiness potential averaged across the mid-frontal region of interest (electrodes F3, Fz, FC1, C3, and Cz) during Adaptation. **B** Mean movement readiness potential averaged across the mid-frontal region of interest during Retention. Shaded error-bars indicate standard error. Black line at 0 ms represents the onset of movement. Voltage (µV) is plotted for Anxiety Early (orange), Control Early (light blue), Anxiety Late (brown), and Control Late (dark blue). Shaded orange area represents the readiness potential, shaded blue area represents the negative slope, and shaded green area represents the motor potential. * represents a main group effect for motor potential (*p* = 0.047). ^ represents a main group effect for negative slope (*p* = 0.033). Plots were created in R using ggplot2 package. Figure aliasing was completed using the geom_smooth defaults

Prior to data synthesis, epoched EEG data was cleaned through trial and ICA removal. The average number of rejected trials (out of 200 total per participant) for the Adaptation condition was 18.74 ± 14.60 (mean ± standard deviation) resulting in 90.06% trials being retained. The number of ICA components removed for this group ranged from 0 to 10 with an average of 4.51 ± 1.14. The average number of rejected trials (out of 200 total per participant) for the Retention condition was 23.90 ± 20.90 resulting in 88.05% trials being retained. The number of ICA components removed for this group ranged from 0 to 3 with an average of 2.17 ± 0.85. Movement readiness potentials for Adaptation and Retention are depicted on Fig. 4.

### Readiness potential

To investigate early preparatory motor activity during the period from − 1500 to − 500 ms before movement onset, we analyzed readiness potential. Readiness potential represents early preparatory activity prior to movement and is generated primarily by the supplementary motor area (SMA), the pre-supplementary motor area (pre-SMA), and cingulate motor area (Cunnington et al. 2003; Kornhuber and Deecke 1965; Praamstra et al. 1996; Shibasaki and Hallett 2006). The analysis revealed no significant differences between Groups, Conditions and Stages. No significant main effects were found for Group (F(1,29.001) = 0.285, *p* = 0.598, *d* = 0.10), Condition (F(1,87.002) = 0.620, *p* = 0.433, *d* = 0.20), or Stage (F(1,87.002) = 2.706, *p* = 0.104, *d* = 0.34). Similarly, no significant interactions emerged: Group × Condition (F(1,87.002) = 2.239, *p* = 0.138, *d* = 0.76), Group × Stage (F(1,87.002) = 0.705, *p* = 0.403, *d =* 0.13), Condition × Stage (F(1,87.002) = 1.080, *p* = 0.302, *d =* 0.61), or Group × Condition × Stage (F(1,87.002) = 0.115, *p* = 0.735, *d = 0.23*). These findings suggest that early preparatory motor activity remains stable across groups and experimental manipulations.

### Negative slope

**Fig. 5:**
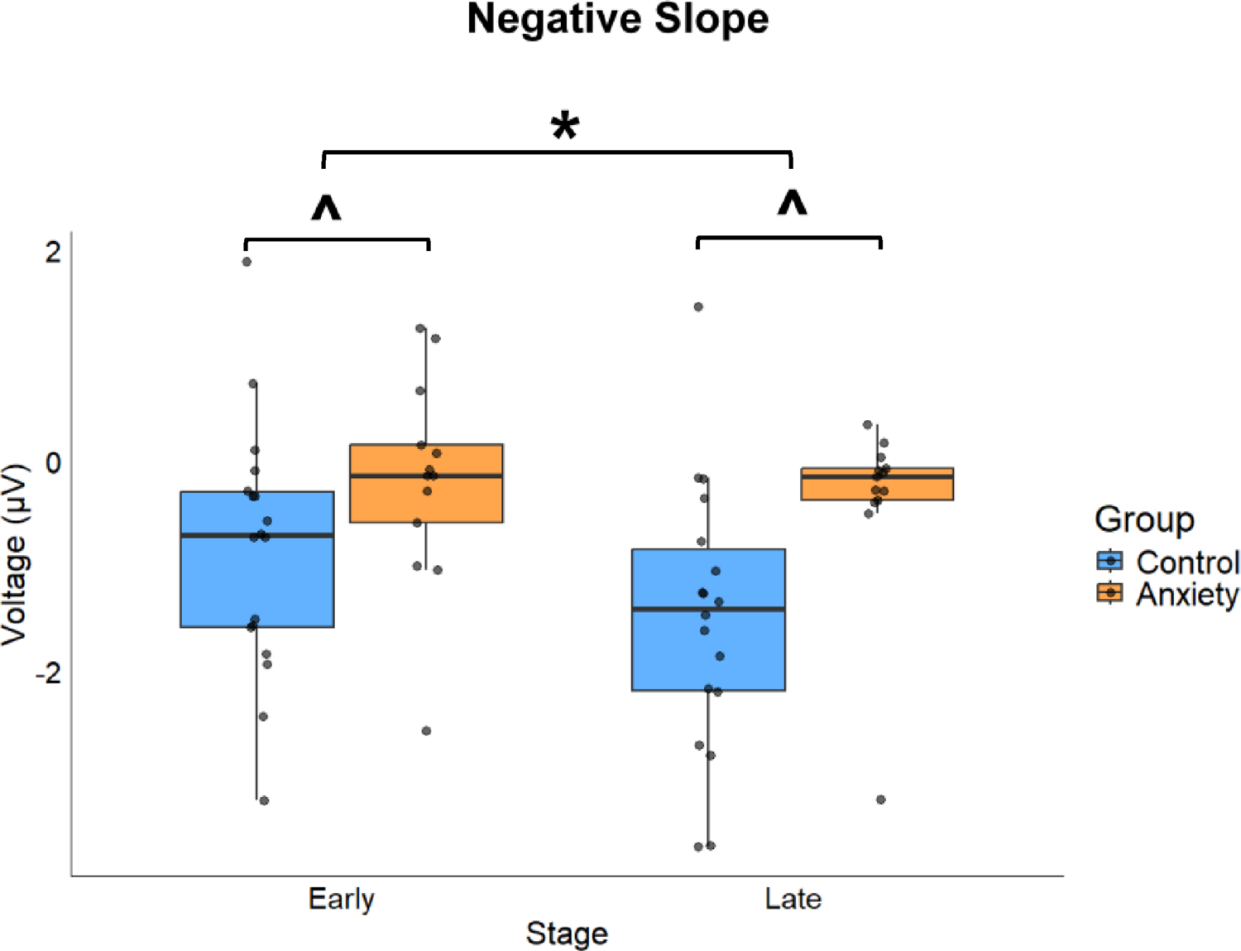
Boxplot for the negative slope component during Early and Late stages across Anxiety and Control groups. Box ends represent the interquartile range (IQR) [25th percentile (Q1) -75th percentile (Q3)]. Whiskers represent minimum (Q1-1.5*IQR) and maximum (Q3–1.5*IQR). Median is represented by horizontal line inside of the box and dots represent individual data. * represents a significant difference from Early (*p* = 0.033). **^** represents a significant difference from Control (*p* = 0.033)

Negative slope represents the translation of movement goals into detailed motor commands (Shibasaki and Hallett 2006; Praamstra et al. 1996; Colebatch, 2007). The anxiety group exhibited reduced preparatory motor activity compared to controls during the late preparatory phase (− 500 to − 100 ms before movement onset) (Fig. 5)>. Significant main effects were found for Group (F(1,29.002) = 5.010, *p*= 0.033, *d =* 0.83) and Stage (F(1,87.000) = 4.697, *p* = 0.033, *d =* 0.31). The anxiety group demonstrated a less negative amplitude compared to the control group [MD: 0.883, *p* = 0.033, 95% CIs = 0.076–1.690, *d =* 0.83]. Early stages showed less negative slopes compared to later stages [MD: 0.423, *p* = 0.033, 95% CIs = 0.037–0.809, *d =* 0.31]. No significant main effect was found for Condition (F(1,87.000) = 0.072, *p* = 0.789, *d =* 0.05). No significant interactions were observed: Group × Condition (F(1,87.000) = 2.563, *p* = 0.113, *d =* 0.30), Group × Stage (F(1,87.000) = 1.478, *p* = 0.227, *d =* 0.39), Condition × Stage (F(1,87.000) = 0.006, *p* = 0.940, *d =* 0.09), or Group × Condition × Stage (F(1,87.000) = 0.145, *p* = 0.704, *d =* 0.25). These findings indicate that anxiety specifically attenuates late-stage motor preparation, with this effect intensifying as movement onset approaches.

### Motor potential

**Fig. 6:**
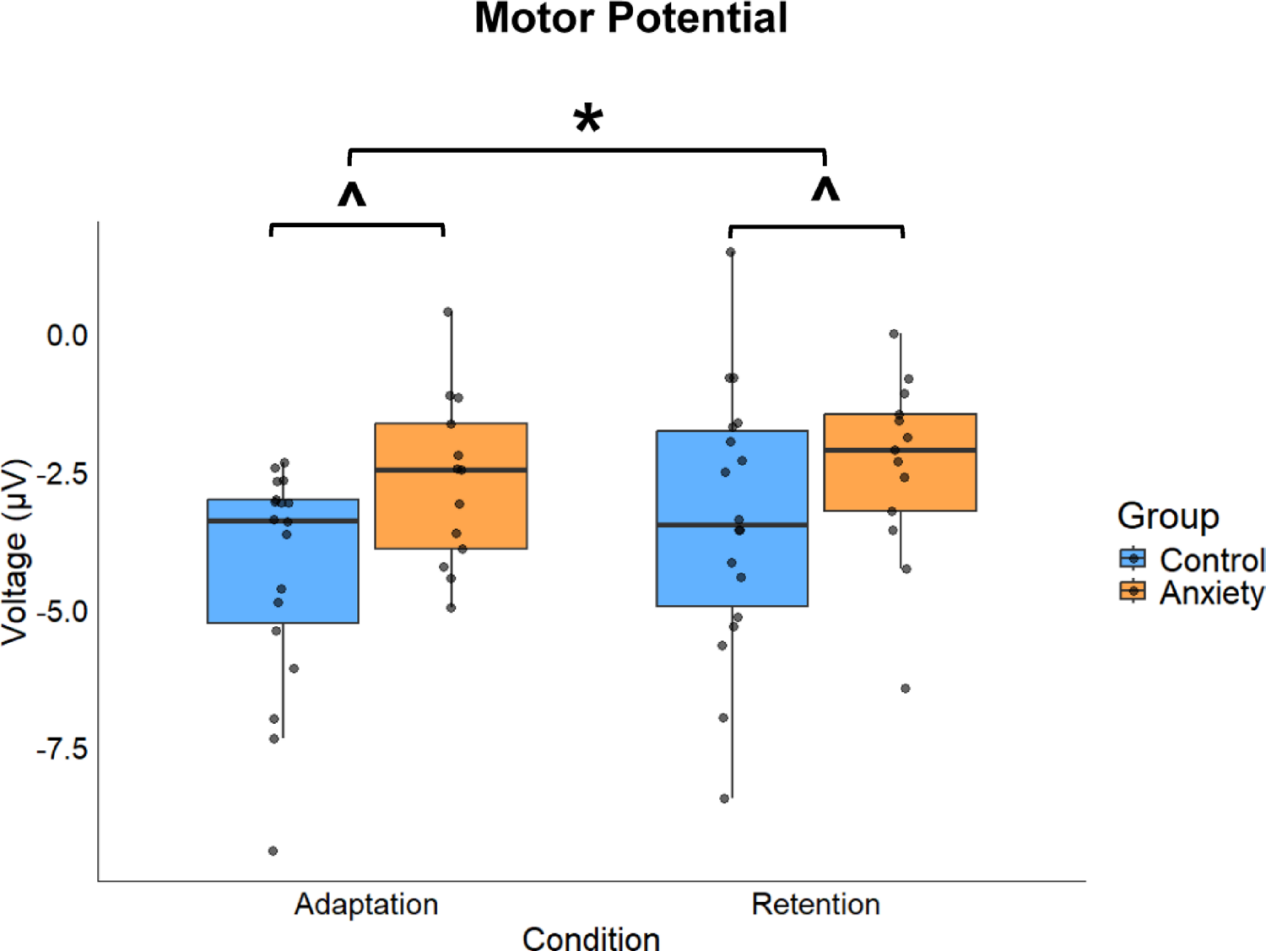
Boxplot for the motor potential component during Adaptation and Retention across Anxiety and Control groups. Box ends represent the interquartile range (IQR) [25th percentile (Q1) -75th percentile (Q3)]. Whiskers represent minimum (Q1-1.5*IQR) and maximum (Q3–1.5*IQR). Median is represented by horizontal line inside of the box and dots represent individual data. * represents a significant difference from Retention (*p* = 0.011). **^** represents a significant difference from Control (*p* = 0.047)

The anxiety group demonstrated reduced motor cortical activation during the movement execution period (− 100 to 100 ms around movement onset) compared to controls, with adaptation phases showing enhanced motor activity overall (Fig. 6). Significant main effects were found for Group (F(1,29.000) = 4.293, *p* = 0.047, *d =* 0.76) and Condition (F(1,87.021) = 6.684, *p* = 0.011, *d =* 0.43). The anxiety group exhibited more positive peak amplitudes compared to the control group [MD: 1.289, *p* = 0.047, 95% CIs = 0.018–2.560]. Adaptation phases elicited more negative peak amplitudes compared to retention phases [MD: 0.598, *p* = 0.011, 95% CIs = 0.143–1.053]. No significant main effect was found for Stage (F(1,87.021) = 0.785, *p* = 0.378, *d =* 0.05). No significant interactions emerged: Group × Condition (F(1,87.021) = 1.973, *p* = 0.164, *d =* 0.06), Group × Stage (F(1,87.021) = 0.360, *p* = 0.550, *d =* 0.31), Condition × Stage (F(1,87.021) = 0.054, *p* = 0.817, *d =* 0.39), or Group × Condition × Stage (F(1,87.021) = 0.103, *p* = 0.749, *d =* 0.24). These amplitude differences suggest that anxiety attenuates motor cortical engagement during voluntary movement execution, and adaptation demands requiring greater motor processing resources compared to retention.

## Discussion

This study evaluated how anxiety disorders change the neural correlates of cognitive-motor integration by examining movement readiness potentials (MRPs) during visuomotor adaptation and retention. Anxious individuals exhibited reduced MRP amplitudes during late motor preparation (− 500 to − 100 ms) and movement execution (− 100 to 100 ms) compared to controls, while early motor preparation (− 1500 to − 500 ms) remained intact, indicating stage-specific disruption. Despite these neural differences, both groups demonstrated equivalent learning and retention. These findings reveal that anxiety disorders selectively disrupt cognitive-motor integration processes, yet compensatory mechanisms preserve skilled learning performance despite neural alterations.

### Anxiety disorders alter late cognitive-motor integration

The movement readiness potential’s late preparatory component, the negative slope, occurs − 500 to − 100 ms before movement onset and reflects translation of movement goals into detailed motor commands (Shibasaki and Hallett 2006; Praamstra et al. 1996; Colebatch, 2007). This component indexes motor specification processes in premotor and primary motor cortices (Cunnington et al., 2002; Jahanshahi et al. 1995; Praamstra et al. 1996), converting movement intentions into precise muscle activations, force profiles, and kinematics (Praamstra et al. 1996; Haggard 2008). The anxiety group exhibited reduced negative slope amplitudes. High-trait anxiety similarly reduces anticipatory frontal activity (Mussini and Di Russo 2023), potentially reflecting reduced preparatory engagement due to heightened uncertainty about responses or outcomes. Movement readiness potentials are strongly modulated by frontal and supplementary motor networks critical for action selection (Brunia et al. 2004; López-Larraz et al. 2014; Di Russo et al. 2017). Reduced negative slope amplitude indicates less efficient motor preparation in anxiety disorders, disrupting motor plan refinement and specification.

Our results align with reduced frontal activity in anxious individuals (Bishop 2009; Ansari and Derakshan 2011). Anxiety disorders show disrupted prefrontal network connectivity, including reduced anterior cingulate cortex (ACC)-dorsomedial prefrontal cortex connectivity in panic disorder (Langhammer et al. 2025), potentially extending to prefrontal-motor pathways. These structural and functional alterations weaken top-down regulation and may specifically disrupt the prefrontal cortex ‘s contribution to late motor preparation. Effective connectivity between the supplementary motor area (SMA) and cingulate cortex sustains motor preparatory activity (Nguyen et al. 2014), suggesting the reduced negative slope we observed reflects disrupted communication within this network. This aligns with literature showing anxiety disrupts prefrontal regions (Kenwood et al. 2021; Roberts and Mulvihill 2024). The prefrontal cortex connects to the SMA primarily through the pre-SMA, which supports cognitive functions, motor selection, and planning (Tanji 1994; Nachev et al. 2008), suggesting prefrontal disruption may impair the motor planning-to-execution transition.

#### Anxiety disorders reduce cortical activity at movement onset

The anxiety group showed lower motor potential amplitudes (− 100 to 100 ms around movement onset) compared to controls. This component reflects primary motor cortex activation immediately before and during movement execution and spinal motoneuron activation(Shibasaki and Hallett 2006; Schurger et al. 2021). The motor cortex receives convergent input from premotor areas, the SMA, and the cerebellum, and relies on this preparatory activity to generate appropriately scaled motor commands (Thach et al. 1992; Tzvi et al. 2020). When preparatory signals are diminished, as we observed in the negative slope component, the motor cortex operates with less robust input, potentially explaining the reduced motor potential amplitude. Recent evidence showed that trait anxiety negatively modulates coupling between motor event-related desynchronization (ERD) and synchronization (ERS) during motor tasks (Cheng et al. 2025). This disrupted coupling suggests anxiety affects both magnitude of motor cortical engagement and coordination between activation-deactivation phases. Reduced MRPs in anxiety align with other psychiatric disorders showing altered motor preparation (Donati et al. 2021; Vöckel et al. 2023), suggesting a transdiagnostic marker of frontal network dysfunction. Both schizophrenia (Luvsannyam et al. 2022) and anxiety (Kenwood et al. 2021) show prefrontal dysfunction affecting voluntary action preparation, potentially reflecting shared prefrontal-motor connectivity vulnerabilities.

### Anxiety disorders do not change early movement preparation

We did not find significant group differences in the readiness potential (− 1500 to − 500 ms prior to movement onset). This early component primarily represents non-lateralized movement preparation generated by the SMA, pre-SMA, and cingulate motor area (Praamstra et al. 1996; Cunnington et al. 2005; Shibasaki and Hallett 2006; Nguyen et al. 2014; Schurger et al. 2021). The lack of group differences in this early stage suggests that the initial preparation for movement is intact in anxiety disorders. Because this component emerges well before conscious awareness of movement (Libet et al. 1983; Cunnington et al. 2005), it reflects a generalized state of motor readiness or excitability rather than the specification of precise movement parameters (Schurger et al. 2021). The prefrontal executive networks that anxiety typically disrupts (Bishop 2007, 2009; Ansari and Derakshan 2011; Eysenck et al. 2023) play a minimal role in this early preparatory stage, suggesting that the readiness potential remains relatively resilient to anxiety-related dysfunction.

#### Visuomotor task progression reduces cortical preparatory activity

Early stages showed smaller amplitudes compared to late stages, consistent with literature showing movement readiness potentials increase within single training sessions due to increases in cortical excitability (Taylor 1978; Staines et al. 2002; Smith and Staines 2012; Jochumsen et al. 2017). While a decrease in amplitude is expected across multiple sessions reflecting improved neural efficiency (Smith and Staines 2012; Jochumsen et al. 2017). Absent Group × Stage interaction indicates anxious individuals show similar learning-related improvements despite consistently lower amplitudes, suggesting preserved capacity to optimize motor preparation with practice despite reduced baseline efficiency.

Adaptation demonstrated greater motor potential than Retention, indicating increased motor cortical engagement when learning versus retaining. This likely reflects the absence of end-point visual feedback during retention. Predictable sensory feedback produces greater movement-related cortical activity than somatosensory feedback alone (Niederberger and Gerber 2006; Reznik et al. 2018; Vercillo et al. 2018; Wen et al. 2018). Importantly, both groups showed this adaptation-retention difference, indicating that anxious individuals retain the capacity to modulate motor cortical activity based on task demands, even though their overall amplitude is reduced. Another possibility for the increase in MRP during adaptation is increased task effort. Our NASA TLX results showcase an overall greater workload while learning to adapt to the 45-degree counterclockwise rotation. Previous studies have noted that high effort under conditions such as fatigue or exertion increases MRP amplitudes (Kristeva et al. 1990; Oda et al. 1996; de Morree et al. 2012; Morree et al. 2014). Thus, the increases in cognitive effort to learn and performed the task increases MRP amplitude, showcasing the integration of cognitive and motor processing during visuomotor adaptation.

#### Influence of pharmaceuticals on cortical dynamics

Medication within the anxiety group represents an important potential source of variability in our movement-related event-related potentials (ERP) measures. A substantial proportion of participants (*n* = 7) were prescribed selective serotonin reuptake inhibitors (SSRIs), which are known to modulate electrophysiological indices of cortical processing (D’Ardhuy et al., 1999). Converging evidence from auditory ERP paradigms indicates that the loudness dependence of auditory evoked potentials is sensitive to monoaminergic function and has been shown to predict antidepressant treatment response, including responsiveness to SSRIs (Linka et al. 2005; Pineles et al. 2023). More broadly, electrophysiological measures such as P300 amplitude and latency have been associated with antidepressant treatment effects and may serve as biomarkers of clinical response, reflecting changes in attentional allocation and stimulus evaluation processes (Bruder et al. 2008; Iosifescu 2011). These findings suggest that pharmacological modulation of serotonergic systems can influence cortical dynamics. A proportion of participants (*n* = 5) were prescribed a dopamine-norepinephrine reuptake inhibitor (DNRI), specifically Bupropion, and were either taking it individually or concurrently with an SSRI or Serotonin-Norepinephrine Reuptake Inhibitor (SNRI). Direct evidence linking DNRI’s to changes in ERP’s is sparse, however a systematic review and meta-analysis by Warren et al. (2023) evaluated the catecholaminergic modulation of ERPs, proposing that dopamine primarily modulates frontally-distributed components including the P3a, N2, and error-related negativity, while norepinephrine modulates the parietally-distributed P3b. Bupropion simultaneously elevates both dopamine and norepinephrine in the prefrontal cortex (Stahl et al. 2004). As such, it is plausible that these effects may extend to motor related processes indexed by the movement readiness potential. However, there is no direct evidence that any of the specific drug classes that the participants were taking directly modulate motor related neural processes. Future studies should further tease out the intricacies of the effects of psychiatric medication on movement preparation.

#### Anxiety disorders alter neural correlates without behavioral impairment

Despite neural differences in motor preparation and execution, anxiety and control groups demonstrated similar behavioral performance throughout the visuomotor adaptation task. Learning curves, retention performance, response times, and peak velocities did not differ between groups, indicating acquisition and recall were unaffected by anxiety disorder status. Weinberg et al. (2010, 2012) demonstrated increased error-related brain activity in individuals with generalized anxiety disorder without observable performance differences, suggesting amplified internal monitoring. A similar pattern of intact behavior with underlying neural changes has been shown in individuals with a history of concussions. Studies consistently report altered electrophysiological responses in the absence of clear behavioral impairments. Specifically, Wilson et al. (2014) showed that contact sport athletes exhibited differences in the P3b component under varying attentional demands without corresponding performance deficits, while Ozen et al. (2013) and Hudac et al. (2018) reported persistent electrophysiological alterations in working memory processes following concussion, even when behavioral performance appeared relatively intact.

The dissociation between reduced MRP amplitudes and intact performance suggests compensatory mechanisms maintain motor learning despite neural inefficiencies (Eysenck et al. 2007; Basten et al. 2011; Berggren and Derakshan 2013). Attentional Control Theory (Eysenck et al. 2007) proposes that anxiety impairs goal-directed attention while increasing stimulus-driven attention and reducing attentional resources. However, compensatory strategies such as increased effort, cognitive monitoring, or alternative processing routes may preserve performance despite neural inefficiencies (Eysenck et al. 2007; Ansari and Derakshan 2011). Shi et al. (2019) meta-analytically confirmed across 58 studies (*N* = 8,92) that anxiety produces reliable deficits in attentional control, including inhibition and attentional switching, with effects on processing efficiency indexed by reaction time. Notably, these deficits were amplified under high cognitive load. In the current study, reaction time did not differ between groups. Given that reaction time serves as a proxy for attentional and cognitive processing efficiency (Khanukhova et al. 2025), this suggests that any additional cognitive burden in the anxiety group was not expressed at the behavioral level. Baker et al. (2011) demonstrated that pre-movement neural activity is directly sensitive to cognitive load, with higher working memory demands reducing movement readiness potential amplitudes in healthy adults. This suggests that attentional and cognitive resources directly modulate motor preparation. The reduction in MRP’s seen in the anxiety group might reflect competition for cognitive control resources. Interestingly, we did not find differences between groups in perceived workload as measured by the NASA-TLX questionnaire. This suggests that the compensatory strategies being used were not consciously perceived as more effortful or attentionally demanding. Another possibility is that the NASA-TLX is not sufficient on its own for assessing subtle differences in attentional control. Similarly, anxiety compromises automatic sensorimotor integration, forcing reliance on effortful conscious control to maintain performance (Nieuwenhuys and Oudejans 2011). Our findings align with both perspectives: although anxiety reduced motor preparation efficiency, as reflected in diminished MRP amplitudes, visuomotor adaptation performance remained comparable across groups, suggesting successful recruitment of compensatory processes.

### Limitations

Although this work provides critical insight into the neural correlates of anxiety disorders, it is not without limitation. We did not test for explicit strategy usage during the task. Previous experiments have showcased the role of explicit aiming strategies to achieve the task goal (Hegele and Heuer 2010; Jahani et al. 2020; Avraham et al. 2021). Another limitation of the study is that the anxiety disorders group were actively taking psychiatric medication to manage their condition. As mentioned above, psychiatric medication may modulate neural activity or behavioral performance (Willerslev-Olsen et al. 2011; Volpato et al. 2016). Importantly, no participant was taking medication that demonstrates active interference with motor cortex functions such as anti-psychotics, anti-epileptics, or barbiturates (Daskalakis et al. 2003; Ziemann et al. 2015). Though our study is comparable to a recent study in this domain, our findings would have benefited from a larger sample size.

## Conclusion

In conclusion, our study provides novel electrophysiological evidence that anxiety disorders alter the brain’s preparation for voluntary movement. Despite these neural differences, behavioral performance remained intact, suggesting that compensatory mechanisms may help preserve motor output in individuals with anxiety. These findings highlight that anxiety disorders impact integrated systems of cognition and action, offering a new perspective on their functional impact. Future research should investigate how acute stress interacts with chronic anxiety to influence motor preparation and performance, as well as explore whether specific anxiety disorder subtypes exhibit distinct neurobehavioral signatures. Given the prefrontal changes seen in this study, future work should use dual-site TMS to directly test prefrontal -motor cortical interactions during movement preparation in individuals with chronic anxiety.

## Data Availability

The raw data supporting the conclusions of this article are available by the authors at [https://osf.io/a65wk].
